# Disrupting differential hypoxia in peripheral veno-arterial extracorporeal membrane oxygenation

**DOI:** 10.1186/s13054-015-0997-3

**Published:** 2015-07-22

**Authors:** Matthew Edward Cove

**Affiliations:** Division of Respiratory and Critical Care Medicine, Department of Medicine, National University Hospital, NUHS Tower Block Level 10, 1E Kent Ridge Road, Singapore, 119228 Singapore

## Abstract

Patients receiving circulatory support with peripheral veno-arterial extracorporeal membrane oxygenation (VA-ECMO) are at risk of developing differential hypoxia. This phenomenon occurs in patients with concomitant respiratory failure. Poorly oxygenated blood, ejected into the ascending aorta from the left ventricle, competes with retrograde flow from the ECMO circuit, potentially causing myocardial and cerebral ischaemia. In a recent *Critical Care* article, Hou et al. use an animal model of peripheral VA-ECMO to study the physiology of differential hypoxia. Their findings support a dual circuit hypothesis, and show how different cannulation strategies can disrupt the two circuits. In particular, strategies that increase venous oxygen saturations in the pulmonary artery can have a large effect on oxygenation saturation in the ascending aorta. The authors provide evidence supporting the use of veno-arterial-venous ECMO in patients who require peripheral VA-ECMO but have simultaneous respiratory failure.

Using peripheral veno-arterial extracorporeal membrane oxygenation (VA-ECMO) for circulatory support, in patients with concomitant respiratory failure, may cause differential hypoxia [1]. Differential hypoxia occurs because patients receiving peripheral VA-ECMO are dependent on retrograde flow, classically from a femoral artery cannula, to deliver oxygenated blood to the upper body. In patients with respiratory failure, however, the left ventricle will eject poorly oxygenated blood into the ascending aorta, which increasingly competes with retrograde flow from the ECMO circuit as cardiac function recovers. If cardiac function is sufficient, poorly oxygenated blood may be preferentially delivered to the myocardium and brain, risking hypoxic injury [2]. Whilst the clinical significance of this phenomenon is not well described, most physicians monitor for differential hypoxia and choose alternative cannulation strategies when it occurs [3].

Differential hypoxia is one of the many reasons why peripheral VA-ECMO should be avoided in patients receiving ECMO for respiratory failure [4], and is also why we should measure arterial haemoglobin oxygen saturation (SO_2_) in both hands of patients receiving peripheral VA-ECMO. Lower saturation readings in the right hand, compared with the left, indicate that this phenomenon is developing. Understanding the physiology of differential hypoxia, and its clinical implications, is important, not least because ECMO use is increasing. According to the Extracorporeal Life Support Organisation (ELSO) January 2015 summary, the reported number of patients receiving ECMO for respiratory failure has more than tripled since 2009, and nearly 15 % of these received some form of VA-ECMO [5].

In a recent *Critical Care* article, Hou et al. [6] added to our understanding of differential hypoxia physiology. By applying peripheral VA-ECMO to an animal model of respiratory failure, with normal cardiac function, Hou et al. created differential hypoxia. Their findings support a dual circuit hypothesis, which magnifies the degree of differential hypoxia. A dual circuit is established when the upper body receives poorly oxygenated blood from the failing native lungs, via the left ventricle, and the lower body receives oxygenated blood from the ECMO circuit. The two circuits are independently sustained because poorly oxygenated blood from the left ventricle supplies only the upper body, drains into the superior vena cava (SVC), and then returns to the left ventricle without being adequately oxygenated. The blood then recycles through the upper body, creating an upper body circuit in which SVC blood is severely deoxygenated. Simultaneously, oxygenated blood from the ECMO circuit perfuses only the lower body and drains into the inferior vena cava (IVC), resulting in a relatively high IVC SO_2_. A separate, well-oxygenated, lower body circuit is maintained because IVC blood returns to the ECMO circuit, via the IVC drainage cannula, and has no opportunity to mix with severely deoxygenated blood from the SVC. The findings of Hou et al. support a dual circuit hypothesis for two reasons. Firstly, contrast introduced into the ECMO circuit does not travel above the level of the diaphragm in the descending aorta, demonstrating that retrograde flow from the ECMO circuit supplies only the lower body. Secondly, they confirm that the two circuits do not mix in the venous circulation, because SO2 in the SVC, pulmonary artery, and ascending aorta is equally poor at 33–40 %, whilst in the IVC it is 84 %.

An important observation in this study was disruption of the two circuits by advancing the IVC cannula into the SVC. As a consequence of this manoeuvre, poorly oxygenated SVC blood preferentially drained into the ECMO circuit, and well-oxygenated venous blood from the IVC returned to the right heart, ultimately reaching the ascending aorta. In Hou et al.’s model, this simple manoeuvre increased SO_2_ in the ascending aorta from 34 to 75 %. Aortic SO_2_ in this range may be sufficient [7]; lower levels are commonly tolerated in climbers, albeit with a long adaptation time [8], and in veno-venous ECMO (VV-ECMO) it is common to target arterial SO_2_ ≧80 %, with no obvious detrimental affects [4]. The authors also show that an additional return cannula, delivering oxygenated blood directly to the internal carotid artery or SVC, further improved SO_2_ in the ascending aorta.

As always, we need to be cautious translating animal findings directly into clinical practice. Firstly, these animals had normal cardiac function at the start of the study. In clinical practice we would not consider VA-ECMO in a patient with normal cardiac function, and normal cardiac function undoubtedly exaggerated differential hypoxia in Hou et al.’s animal model. Whilst this makes the physiology easier to study, it may also exaggerate the potential benefits of modifying cannulation strategies. Secondly, although carotid cannulation has been commonly used in paediatric ECMO, the potential neurological sequelae could be considerable in adults with existing carotid artery disease. Finally, the safety of advancing a cannula from the IVC to the SVC is not established, although modern double lumen catheters, advanced from the SVC to the IVC, are in clinical use with VV-ECMO [9].

Despite these concerns, the findings from this animal study can still be applied to clinical practice. Demonstrating improved SO_2_ in the ascending aorta, simply by adding an additional cannula returning oxygenated blood to the SVC, supports the use of veno-arterial-venous ECMO (VAV-ECMO) in patients who require VA-ECMO but have concomitant respiratory failure. VAV-ECMO describes an ECMO cannulation strategy where oxygenated blood is returned to both the arterial system and the venous system, typically via the femoral artery and the SVC (right internal jugular vein cannula), respectively [10].

In conclusion, Hou et al. have elegantly demonstrated that a dual circuit mechanism may be responsible for sustaining differential hypoxia in patients receiving peripheral VA-ECMO. They have also shown that simply draining blood from the SVC, or returning oxygenated blood to the SVC and proximal arterial circulation, disrupts the two circuits and delivers adequately oxygenated blood into the ascending aorta. Therefore, when faced with a patient requiring mechanical circulatory support, who also has severe respiratory failure, VAV-ECMO would seem a logical approach—certainly based on the animal model presented by Hou et al.

## Abbreviations

ECMO: Extracorporeal membrane oxygenation
ELSO: Extracorporeal Life Support Organisation
IVC: Inferior vena cava
SO_2_: Oxygen saturation
SVC: Superior vena cava
VA-ECMO: Veno-arterial extracorporeal membrane oxygenation
VAV-ECMO: Veno-arterial-venous extracorporeal membrane oxygenation
VV-ECMO: Veno-venous extracorporeal membrane oxygenation
